# Long-axial field-of-view PET/CT for the assessment of inflammation in calcified coronary artery plaques with [^68^ Ga]Ga-DOTA-TOC

**DOI:** 10.1007/s00259-023-06435-6

**Published:** 2023-09-23

**Authors:** Clemens Mingels, Hasan Sari, Nasir Gözlügöl, Carola Bregenzer, Luisa Knappe, Korbinian Krieger, Ali Afshar-Oromieh, Thomas Pyka, Lorenzo Nardo, Christoph Gräni, Ian Alberts, Axel Rominger, Federico Caobelli

**Affiliations:** 1 Department of Nuclear Medicine, Inselspital, University Hospital Bern, University of Bern, Freiburgstr. 18, 3010 Bern, Switzerland; 2 Advanced Clinical Imaging Technology, Siemens Healthcare AG, Lausanne, Switzerland; 3 Department of Radiology, University of California Davis, Davis, CA USA; 4 Department of Cardiology, Inselspital, University Hospital Bern, University of Bern, Bern, Switzerland; 5 Molecular Imaging and Therapy, BC Cancer Agency, 600 West 10th Ave, Vancouver, BC V5Z 1H5 Canada

**Keywords:** Whole-body PET/CT, LAFOV PET/CT, Inflamed coronary plaques, Atherosclerosis, Somatostatin receptor imaging

## Abstract

**Purpose:**

Inflamed, prone-to-rupture coronary plaques are an important cause of myocardial infarction and their early identification is crucial. Atherosclerotic plaques are characterized by overexpression of the type-2 somatostatin receptor (SST_2_) in activated macrophages. SST_2_ ligand imaging (e.g. with [^68^ Ga]Ga-DOTA-TOC) has shown promise in detecting and quantifying the inflammatory activity within atherosclerotic plaques. However, the sensitivity of standard axial field of view (SAFOV) PET scanners may be suboptimal for imaging coronary arteries. Long-axial field of view (LAFOV) PET/CT scanners may help overcome this limitation. We aim to assess the ability of [^68^ Ga]Ga-DOTA-TOC LAFOV-PET/CT in detecting calcified, SST_2_ overexpressing coronary artery plaques.

**Methods:**

In this retrospective study, 108 oncological patients underwent [^68^ Ga]Ga-DOTA-TOC PET/CT on a LAFOV system. [^68^ Ga]Ga-DOTA-TOC uptake and calcifications in the coronary arteries were evaluated visually and semi-quantitatively. Data on patients’ cardiac risk factors and coronary artery calcium score were also collected. Patients were followed up for 21.5 ± 3.4 months.

**Results:**

A total of 66 patients (61.1%) presented with calcified coronary artery plaques. Of these, 32 patients had increased [^68^ Ga]Ga-DOTA-TOC uptake in at least one coronary vessel (TBR: 1.65 ± 0.53). Patients with single-vessel calcifications showed statistically significantly lower uptake (SUV_max_ 1.10 ± 0.28) compared to patients with two- (SUV_max_ 1.31 ± 0.29, *p* < 0.01) or three-vessel calcifications (SUV_max_ 1.24 ± 0.33, *p* < 0.01). There was a correlation between coronary artery calcium score (CACS) and [^68^ Ga]Ga-DOTA-TOC uptake, especially in the LAD (*p* = 0.02). Stroke and all-cause death occurred more frequently in patients with increased [^68^ Ga]Ga-DOTA-TOC uptake (15.63% vs. 0%; *p*:0.001 and 21.88% vs. 6.58%; *p*: 0.04, respectively) during the follow-up period.

**Conclusion:**

[^68^ Ga]Ga-DOTA-TOC as a marker for the macrophage activity can reveal unknown cases of inflamed calcified coronary artery plaques using a LAFOV PET system. [^68^ Ga]Ga-DOTA-TOC uptake increased with the degree of calcification and correlated with higher risk of stroke and all-cause death. [^68^ Ga]Ga-DOTA-TOC LAFOV PET/CT may be useful to assess patients’ cardiovascular risk.

**Supplementary Information:**

The online version contains supplementary material available at 10.1007/s00259-023-06435-6.

## Introduction

In patients with coronary artery disease (CAD), inflamed and prone-to-rupture coronary plaques are associated with higher risk of major adverse cardiac events (MACE) [1]. Accordingly, numerous invasive and non-invasive approaches for their early identification and characterization have been tested. Among various possible imaging targets, macrophage infiltration, especially sustained by pro-inflammatory monocyte-derived macrophages (M1-phenotype), have emerged as a potential marker of plaque vulnerability [1, 2]. In fact, lipid-derived metabolites such as low-density lipoprotein (LDL), that can be found in coronary plaques, are known to stimulate the migration of macrophages into the arterial intima, wherein they mature and become phagocytic [2, 3]. These “activated” macrophages then upregulate inflammatory metabolic pathways causing the progression of CAD [1, 4].

Positron emission tomography (PET) is an excellent tool for the assessment and the characterization of various metabolic processes. Metabolic radiotracers like [^18^F]FDG proved reliable in the evaluation of inflamed plaque [5], but lacks specificity for the identification of activated macrophages. In this regard, somatostatin receptor 2 (SST_2_) imaging may represent an important advance, as SST_2_, a G-protein-coupled transmembrane protein, is overexpressed by M1-macrophages [6, 7]. Consistent with this concept, it was reported that SST_2_ imaging yields improved accuracy in discriminating high-risk versus low-risk coronary lesions than [^18^F]FDG [8, 9].

While SST_2_ imaging with PET has proven promising in the evaluation of inflamed vascular plaque in patients investigated for oncological reasons [10, 11], its wider implementation has been precluded by its lower diagnostic accuracy compared to [^18^F]FDG as a result of its lower sensitivity. The relatively low total number of activated macrophages in plaques causes low signal-to-noise ratio (SNR) in SST_2_ imaging, whereas [^18^F]FDG is imported by glucose transporters (GLUT), which are upregulated on a wide variety of inflammatory cells and not limited to macrophages [12, 13]. Other potential challenges include motion artifacts (cardiac and respiratory), shorter half-life (68 min vs. 110 min) and lower positron yield of the ^68^ Ga compared to for example ^18^F [14]. Hence, areas affected by an infiltration of M1-macrophages are difficult to image on conventional PET scanners due to low signal collection efficiency and limited resolution [15, 16]. This gap has now been closed with the introduction of new silicon photomultiplier (SiPM)-based, long-axial field-of-view (LAFOV) PET/CT scanners. The recent clinical implementation of LAFOV PET/CT with 15 fold improvement in sensitivity and a spatial resolution of approximately 3 mm allows for identification and quantification of small areas with low radiotracer uptake [17–21]. The higher sensitivity of LAFOV systems results in higher temporal resolution [22]. This could be of utility in gated acquisitions and facilitate the imaging of structures vulnerable to motion artifacts, such as the coronary arteries.

This study aims to evaluate the detectability of calcified coronary artery plaques overexpressing SST_2_ on LAVOF PET scanners. To investigate SST_2_ overexpression as marker of plaque vulnerability, PET findings were correlated to cardiovascular risk factors and clinical outcomes.

## Materials and methods

### Patient population and clinical information

This is a single-center, retrospective observational study collecting data from a cohort of oncologic patients (*n* = 113) who underwent clinical routine [^68^ Ga]Ga-DOTA-TOC (DOTA = tetraazacyclododecane tetraacetic acid and TOC = D-Phe-c(Cys-Tyr-D-Trp-Lys-Thr-Cys)-Thr(ol)) PET/CT scans between January 2021 and December 2021 on a LAFOV PET/CT [23]. Patients with known coronary artery disease were excluded for the analysis (*n* = 5). Electronic medical records were searched for the presence of established cardiovascular risk factors [24] (Table 1). Clinical records of MACE (myocardial infarction, hospitalization for cardiac reasons, stroke, coronary artery revascularization) and/or all-cause death during the follow-up period were also collected (Table 2). Patients were followed up for 21.5 ± 3.4 months. Patients’ characteristics are outlined in the Supplementary Material (Table 1).

**Table 1:** A total of 108 patients received [^68^ Ga]Ga-DOTA-TOC PET/CT of which 66 showed calcified coronary artery plaques. Outlined are patients’ characteristics for both subgroups (either in percentages (%) or in mean ± SD) in both subgroups (with and without [^68^ Ga]Ga-DOTA-TOC uptake)

	Overall (*n* = 108)			Subgroub: patients with calcified coronary arteries (*n* = 66)		
Risk factors	With [^68^ Ga]Ga-DOTA-TOC uptake (*n* = 32)	Without [^68^ Ga]Ga-DOTA-TOC uptake (*n* = 76)	*p*-value	With [^68^ Ga]Ga-DOTA-TOC uptake (*n* = 32)	Without [^68^ Ga]Ga-DOTA-TOC uptake (*n* = 34)	*p*-value
Hypercholesterolaemia	15.63%	15.79%	1.00	15.63%	20.59%	0.75
LDL [mmol/L]	2.33 ± 1.34	2.61 ± 0.88	0.63	2.33 ± 1.34	2.40 ± 0.94	0.22
Arterial hypertension	34.38%	25.00%	0.35	34.38%	35.29%	1.00
History of smoking	0%	2.63%	1.00	0%	2.94%	1.00
Diabetes mellitus (type II)	6.25%	9.21%	0.67	6.25%	11.76%	0.67
Family history of heart disease	0%	2.63%	1.00	0%	2.94%	1.00
Prior cardio-vascular events	3.13%	2.63%	1.00	3.13%	2.94%	1.00
Peripheral artery disease (PAD)	0%	1.32%	1.00	0%	0%	1.00
Follow-up [month]	22.94 ± 3.13	20.82 ± 3.3	0.95	22.94 ± 3.13	21.62 ± 2.88	0.97

*P*-values < 0.05 are considered statistically significant, indicated by an asterisk (“*”)

**Table 2:** Major adverse cardiac events (MACE) during follow up overall and for patients with calcified coronary artery plaques. Outlined are patients’ characteristics for both subgroups (either in percentages (%) or in mean ± SD) in both subgroups (with and without [^68^ Ga]Ga-DOTA-TOC uptake)

	Overall (*n* = 108)			Subgroub: patients with calcified coronary arteries (*n* = 66)		
MACE during follow-up	With [^68^ Ga]Ga-DOTA-TOC uptake (*n* = 32)	Without [^68^ Ga]Ga-DOTA-TOC uptake (*n* = 76)	*p*-value	With [^68^ Ga]Ga-DOTA-TOC uptake (*n* = 32)	Without [^68^ Ga]Ga-DOTA-TOC uptake (*n* = 34)	*p*-value
Stroke	15.63%	0%	0.001 (*)	15.63%	0%	0.02 (*)
Myocardial Infarction	0	0	1.00	0	0	1.00
Hospitalization for cardiac reasons	0	0	1.00	0	0	1.00
Coronary artery re-vascularization	0	0	1.00	0	0	1.00
Death	21.88%	6.58%	0.04 (*)	21.88%	8.82%	0.18

*P*-values < 0.05 are considered statistically significant, indicated by an asterisk (“*”)

### Imaging protocol

PET images were acquired 60 min after intravenous injection of 152.2 ± 9.2 MBq [^68^ Ga]Ga-DOTA-TOC on a LAFOV PET/CT scanner (Biograph Vision Quadra, Siemens Healthineers, Erlangen, Germany). Images were acquired in list-mode for 10 min in a single-bed position (skull-vertex to mid femur). Image reconstruction was performed as previously described using high sensitivity mode (HS, (MRD) maximum ring difference of 85) [18]. The used MRD enabled a uniform sensitivity profile across the axial FOV [19]. Non-contrast enhanced, low-dose CT images were used for attenuation correction and to identify and score calcified coronary artery plaques [25]. CT characteristics were published previously [26, 27]. The same CT acquisitions were used to assess coronary artery calcium score (CACS).

### Image evaluation

Two nuclear medicine physicians independently from each other evaluated all images. Appropriate workstations and software were used for quantitative image analysis and identification of target lesions (Syngo.via, Siemens Healthineers, Erlangen, Germany) [28].

Standardized uptake values (SUV_max/peak_) of calcified plaques were assessed by manually placing a volume-of-interest (VOI) with a 40%-iso-contour around the lesion, as previously described [26, 29]. For the assessment of background activity (expressed as SUV_mean_), 10 cm^3^ VOI were manually drawn in the descending aorta (blood pool). Target-to-background ratio (TBR) was calculated as the ratio of SUV_max_ of the calcified plaque and the mediastinal blood pool (SUV_mean_). A plaque was defined as [^68^ Ga]Ga-DOTA-TOC-avid with visual detectable uptake if TBR was > 1.00.

### Patients grouping

Patients were subdivided in two groups according to the presence of calcified plaques. Patients with calcified coronary plaques were further divided into patients with and without [^68^ Ga]Ga-DOTA-TOC uptake. Patients with calcified plaques were also grouped according to the number of affected coronary vessels (score 1–3). CACS was calculated using the Syngo.via Calcium-Score tool according to Agatston, with a threshold of 130 Hounsfield Units (HU) as previously described [25, 30].

### Statistical analysis

Statistical analysis was performed using Graphpad Prism Version 8 (San Diego, California). Data are presented either as mean ± standard deviation (SD) or as median and range. Comparisons between different groups (with/without coronary calcification, with/without [^68^ Ga]Ga-DOTA-TOC uptake) were performed using Fisher’s exact test for proportions and with Student’s *T*-test after testing for normal distribution applying Kolmogorov–Smirnov test for continuous values. Correlation between [^68^ Ga]Ga-DOTA-TOC uptake and calcifications was tested either using Pearson’s correlation coefficient or using a linear correlation model. Uptake changes after peptide receptor radionuclide therapy (PRRT) in semi-quantitative image parameters (e.g., SUV) were characterized using paired Student’s *T*-test. *P* values < 0.05 were considered statistically significant.

## Results

### Calcified coronary artery plaques and patient-based sensitivity

A total of 108 patients were included in the analysis, without known coronary artery disease (CAD). Of these, 66 patients (61.1%) had calcified plaques of any degree in the coronary arteries. Calcified plaques were detected in one single vessel in 30/66 patients (45.5%), in two vessels in 12/66 (18.2%), and in three vessels in 24/66 (36.4%). Mean CACS was 21.0 (1.8–271.0). Most affected coronary arteries were left anterior descending artery (LAD 18.40 (IQR 0–192.6), right coronary artery (RCA) 3.8 (0-IQR 69.9), left main (LM) 0 (IQR0-36.2), and left circumflex artery (LCX) 0 (IQR0-28.0)), respectively.

**Fig. 1:**
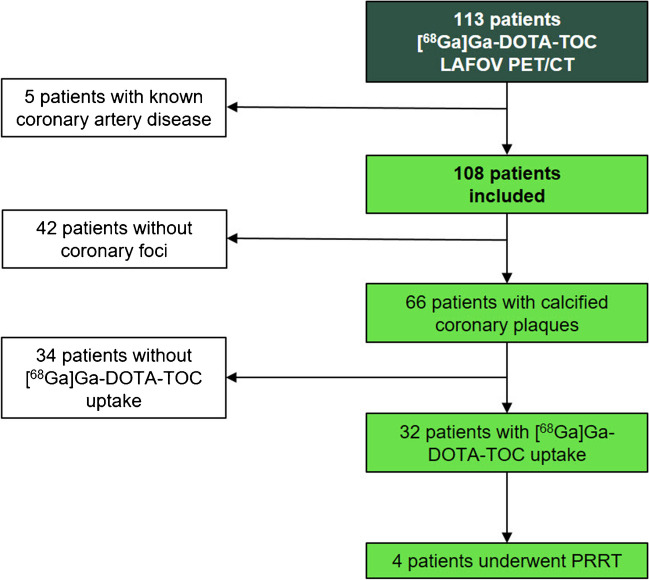
Study flowchart. Study flowchart showing patient selection and included patients. LAFOV, long-axial field-of-view; PRRT, peptide receptor radionuclide therapy

**Fig. 2:**
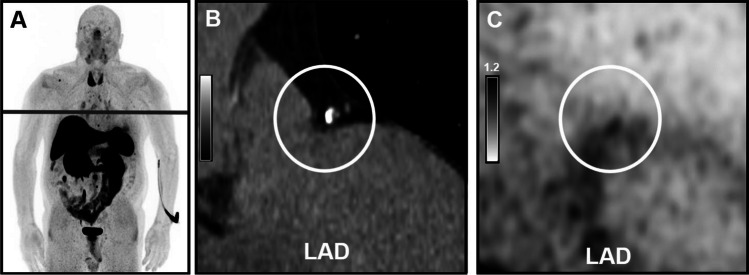
Example image revealing [^68^ Ga]Ga-DOTA-TOC uptake in calcified LAD plaque. A 78 y/o male patient with neuroendocrine tumor (NET) of the ileum was referred for [^68^ Ga]Ga-DOTA-TOC LAFOV PET/CT. An unknown calcified plaque with significant [^68^ Ga]Ga-TOTA-TOC uptake (SUV_max_: 1.75, TBR: 2.50) was detected in the LAD. **A** shows the maximum intensity projection (MIP), **B** shows a coronal CT image, and **C** shows the coronal PET image

Among patients with detectable calcified plaques, increased [^68^ Ga]Ga-DOTA-TOC uptake was present in at least one coronary artery in 32 patients (48.48%), with SUV_max_ 1.21 ± 0.30, SUV_peak_ 1.01 ± 0.23, TBR 1.65 ± 0.53 (Figs. 1, 2). In patients with calcifications, increased [^68^ Ga]Ga-DOTA-TOC uptake was found in in the LM in 6%, in LAD in 69%, in LCX in 16% and in RCA in 9%. No patients had focally increased [^68^ Ga]Ga-DOTA-TOC uptake in a coronary artery without corresponding calcified plaque.

Global and single-vessel CACS for all 108 patients were compared with regard to the [^68^ Ga]Ga-DOTA-TOC uptake*.* Patients were divided into two groups (with and without uptake). Global and single-vessel CACS was significantly higher in the subgroup with coronary [^68^ Ga]Ga-DOTA-TOC uptake (130.4 (20.00–509.50) vs. 0 (0–17.30), *p*: < 0.01). Global and single-vessel CACS are outlined in Table 3.

**Table 3:** One hundred eight patients received [^68^ Ga]Ga-DOTA-TOC PET/CT. Shown are the Calcium Scores (median and range) or the left main (LM), left anterior descending (LAD), left circumflex (LCX), right coronary artery (RCA), and global calcium score in both subgroups (with and without coronary tracer uptake)

Calcium score (*n* = 108)	With coronary [^68^ Ga]Ga-DOTA-TOC uptake (*n* = 32)	Without coronary [^68^ Ga]Ga-DOTA-TOC uptake (*n* = 76)	*p*-value
LM	1.3 (0–43.20)	0 (0–0)	0.02 (*)
LAD	36.9 (0.95–229.80)	0 (0–5.88)	0.02 (*)
LCX	3.2 (0–31.15)	0 (0–0)	0.05 (*)
RCA	11.4 (0–191.05)	0 (0–0)	0.01 (*)
**Global**	**130.4 (20.00–509.50)**	**0 (0–17.30)**	< 0.01 (*)

*P*-values < 0.05 are considered statistically significant, indicated by an asterisk (“*”)

### Correlation of [^68^ Ga]Ga-DOTA-TOC uptake and calcification

There was a significant correlation between CACS and [^68^ Ga]Ga-DOTA-TOC uptake (*p* = 0.03 for SUV_max_ and *p* = 0.04 for SUV_peak_).

**Fig. 3:**
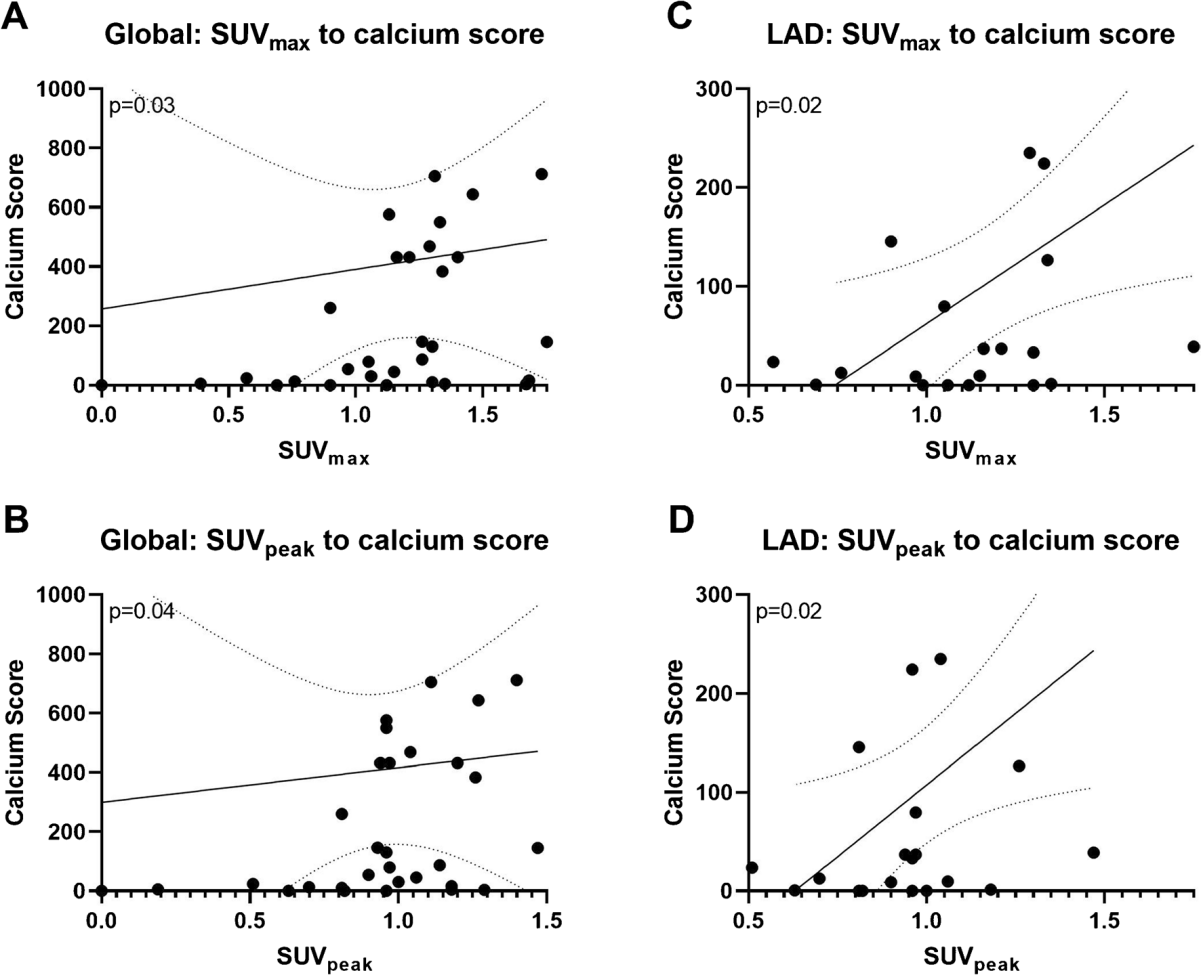
Linear correlation of Calcium Score and SUV_max/peak_. Indicated are the linear regression models (best fit and standard deviations) of global (**A**/**B**) and LAD (**C**/**D**) calcium score to SUV_max_ (**A**/**C**) and SUV_peak_ (**B**/**D**); *p*-values are given

Sub-analysis of the relationships between CACS in each coronary vessel and [^68^ Ga]Ga-DOTA-TOC uptake showed a significant, positive correlation in LAD (SUV_max_ *p*: 0.02 and SUV_peak_ *p*:0.02) (Fig. 3). Conversely, there was no significant correlation in LM, LCX and RCA for both SUV_max_ and SUV_peak_.

**Fig. 4:**
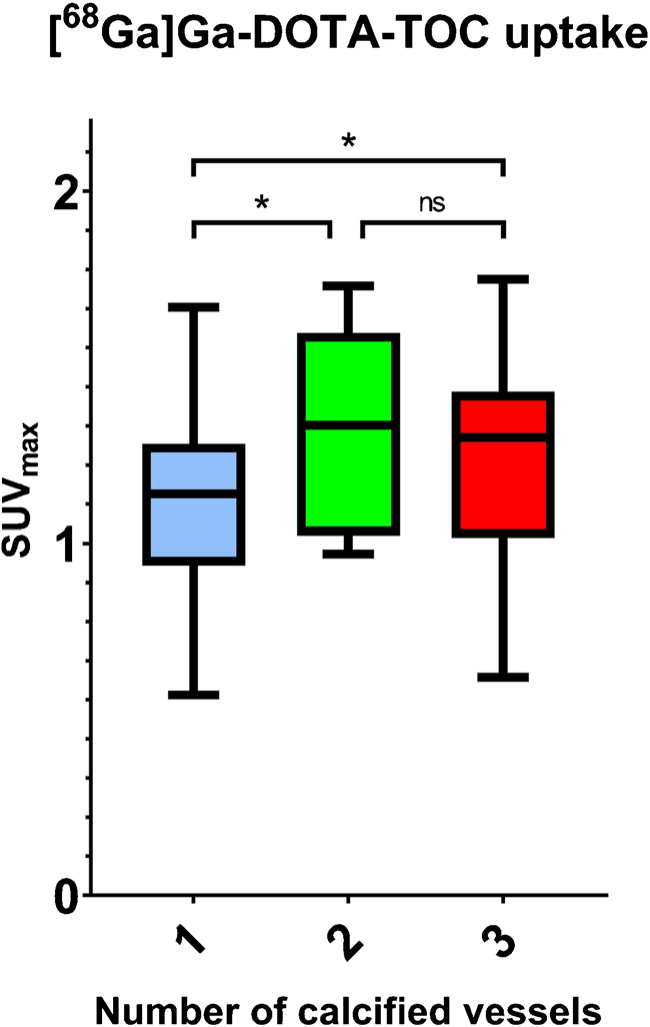
[^68^ Ga]Ga-DOTA-TOC uptake by the number of calcified vessels. Shown is the [^68^ Ga]Ga-DOTA-TOC uptake by the number of calcified vessels. Patients showed one, two, or three calcified coronary arteries (number of calcified vessels 1–3) and were grouped accordingly. Patients with ≥ 2 affected coronary arteries showed significantly higher tracer uptake (“*”) characterized by SUV_max_

Patients with single-vessel calcifications showed lower [^68^ Ga]Ga-DOTA-TOC uptake (SUV_max_: 1.10 ± 0.34; SUV_peak_: 0.90 ± 0.25) compared to patients with two- (SUV_max_: 1.31 ± 0.34, SUV_peak_: 1.03 ± 0.24, *p* < 0.01) or three-vessel calcifications (SUV_max_: 1.21 ± 0.26, SUV_peak_: 1.02 ± 0.22, *p* < 0.01) (Fig. 4). Conversely, the localisation of the calcified plaques in different vessels did not correlate with a different degree of [^68^ Ga]Ga-DOTA-TOC uptake (Supplementary Material). However, unlike LAD, the frequency of calcified plaques with [68 Ga]Ga-DOTA-TOC uptake was low (LM *n* = 2, RCX *n* = 5, RCA *n* = 3).

### Correlation with cardiac risk factors

Patient-specific cardiovascular risk factors (e.g., hypercholesterolaemia, hypertension, smoking, diabetes, family history of heart diseases, prior cardiovascular events, peripheral artery disease (PAD), and prior stroke) for an occurrence of a major cardiac event were collected. Patients with calcified coronary plaques more frequently had hypertension compared to patients without sclerotic lesions (34.9% vs. 16.7%, *p*: 0.04). Furthermore, there was a tendency toward higher proportion of hypercholesterolemia (18.2% vs. 11.9%) and prior history of stroke (7.6% vs. 0%) in patients with calcified coronary plaques, however without statistical significance (*p* = 0.43 and 0.15, respectively).

### Follow-up

Patients with increased [^68^ Ga]Ga-DOTA-TOC uptake of the calcified coronary plaques had higher rate of all-cause death (21.9% vs. 6.6%; *p*: 0.04) as well as stroke (15.6% vs. 0%; *p*:0.001) compared to patients without (Table 2). Subgroup analysis of patients with calcified coronary arteries showed that stroke during the follow-up period occurred significantly higher (15.6% vs. 0%; *p*: 0.02), whereas no significant difference in the appearance of all-cause death was seen between patients with and without [^68^ Ga]Ga-DOTA-TOC plaque uptake.

Overall, 12 patients died during the follow-up period. A total of 3/12 suffered from organ failure; cause of death for 9/12 was not known.

### [^68^ Ga]Ga-DOTA-TOC uptake before and after PRRT

**Fig. 5:**
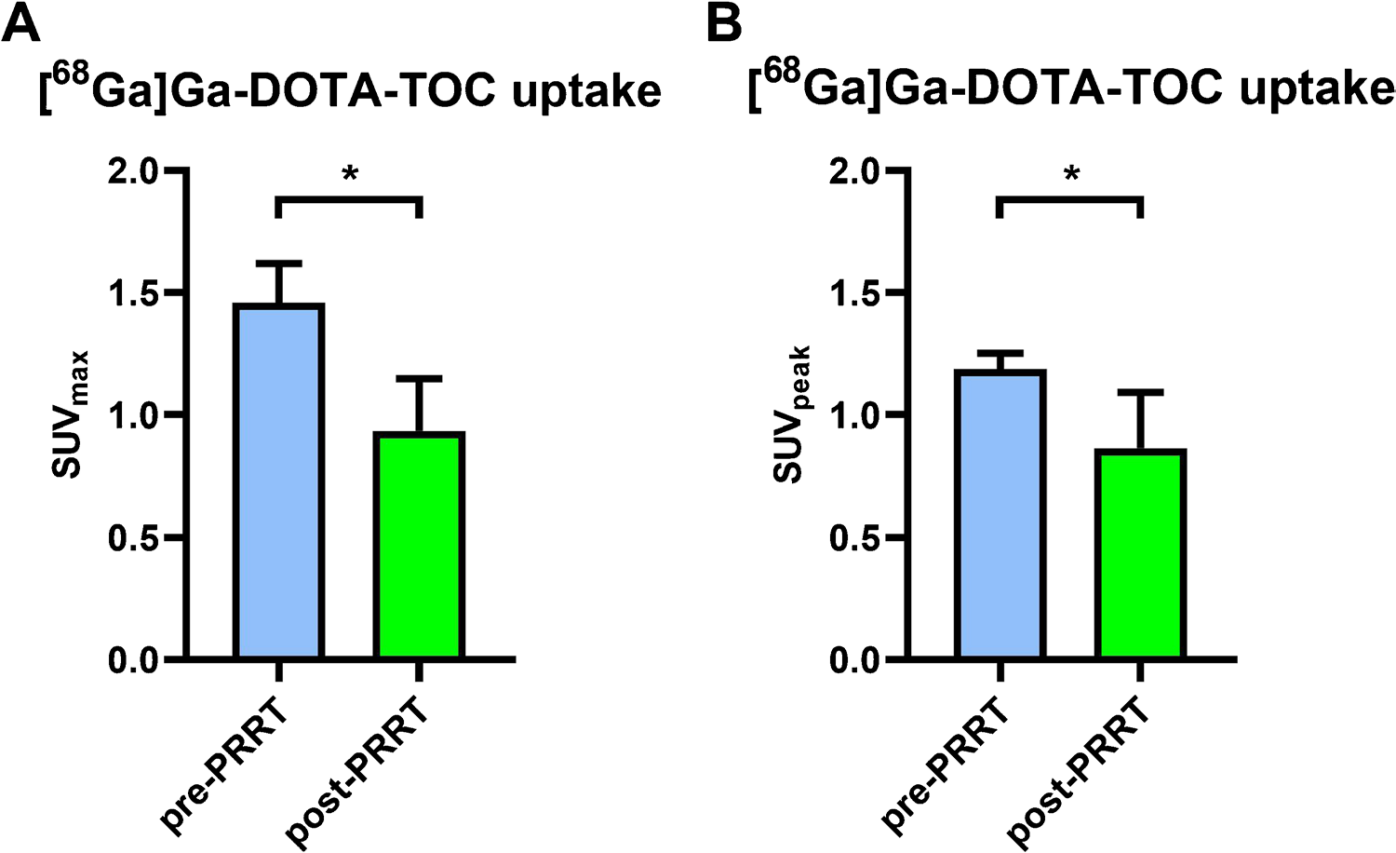
[^68^ Ga]Ga-DOTA-TOC uptake before and after PRRT. Shown is the [^68^ Ga]Ga-DOTA-TOC uptake in coronary artery plaques, SUV_max_ (**A**) and SUV_peak_ (**B**) before (blue) and after (green) peptide receptor radionuclide therapy (PRRT). Seen was a significant degrees of [.^68^ Ga]Ga-DOTA-TOC uptake (“*”)

**Fig. 6:**
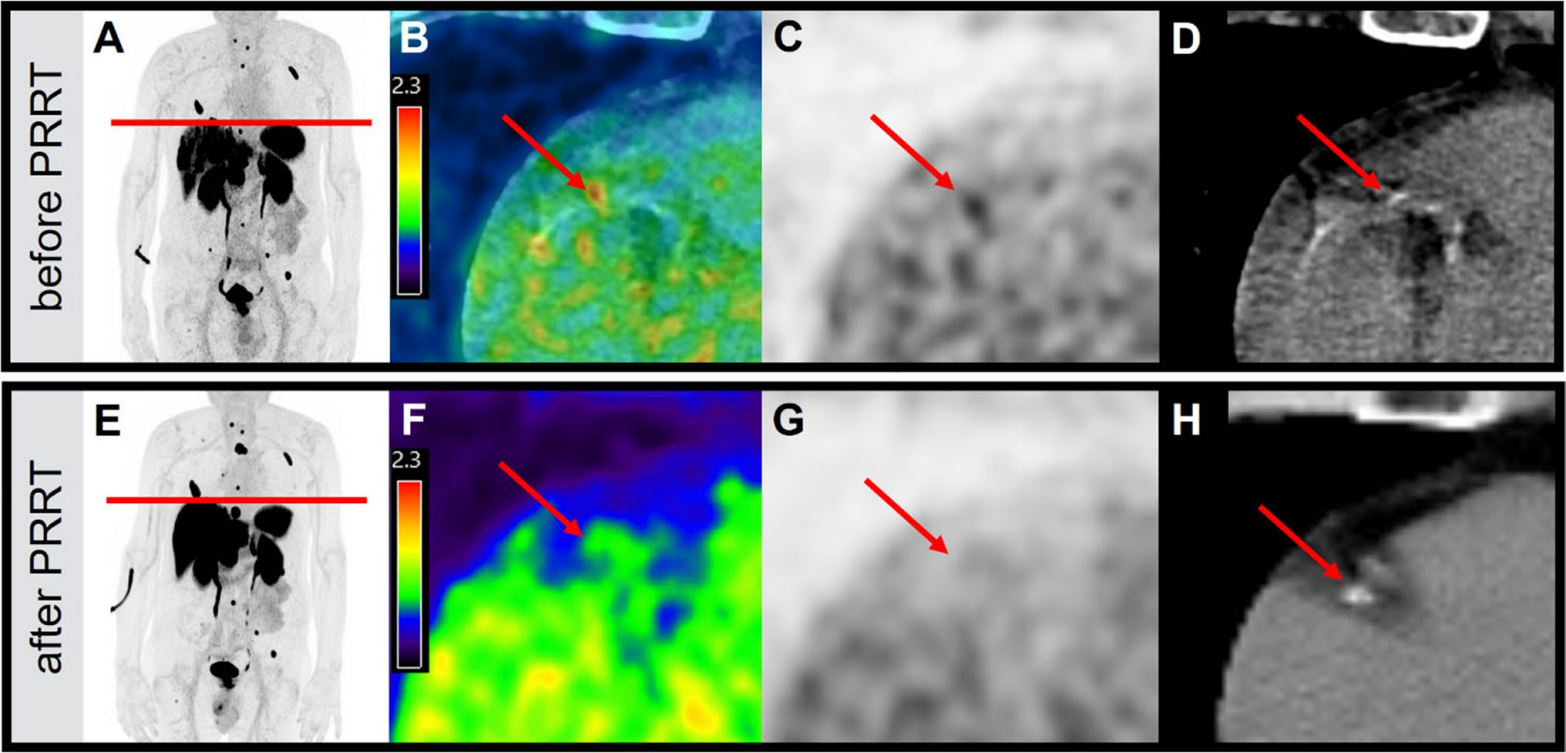
[^68^ Ga]Ga-DOTA-TOC uptake before and after PRRT. A 80 y/o male patient with metastatic neuroendocrine tumor of the pancreas was treated with peptide receptor radionuclide therapy (PRRT). Shown are maximum intensity projections (MIP – **A**/**E**), axial fusion images (**B**/**F**), stand alone PET (**C**/**G**) and stand-alone CT (**D**/**H**) images of a calcified inflamed RCA plaque (marked with a red arrow). **A**–**D** show the [^68^ Ga]Ga-DOTA-TOC uptake before and **E**–**H** after PRRT

Four of 108 patients (3.7%) underwent PRRT. Coronary [^68^ Ga]Ga-DOTA-TOC uptake at LAFOV PET/CT pre- and post PRRT was compared. Semi-quantitative analysis (SUV_max/peak_) revealed decreased tracer uptake in the calcified coronary artery plaques after PRRT (SUV_max_: 1.46 ± 0.14 vs. 0.94 ± 0.19, *p*: 0.01 and SUV_peak_: 1.19 ± 0.06 vs. 0.87 ± 0.20, *p*: 0.03) (Fig. 5). Two patients showed significant [68 Ga]Ga-DOTA-TOC uptake in calcified plaques within LAD (Ca-Score range: 5.1–351.6) and 2 within RCA (Ca-Score range: 16.5–218.1). None of these patients had prior history of cardiac disease. Representative images of [^68^ Ga]Ga-DOTA-TOC uptake pre- and post PRRT are outlined in Fig. 6.

## Discussion

In this study, we report the first data for [^68^ Ga]Ga-DOTA-TOC imaging to detect inflamed calcified coronary artery plaques using a LAFOV PET/CT system. Previous work indicated that PET-based SST_2_ imaging has a clear potential in the detection of inflamed vascular plaques, with a more specific affinity compared to [^18^F]FDG [8, 9], but to-date, the detection of SST_2_ positive calcified plaques in routine PET/CT has been challenging. Li et al. report increased [^68^ Ga]Ga-DOTA-TATE uptake in 4/16 patients with calcified plaques within large arteries, resulting in a detection rate of 25% [31]. The detection and quantification of [^68^ Ga]Ga-DOTA-TOC uptake in small vessels such as the coronary arteries has been even more challenging due to intrinsic, relative limitations in spatial and temporal resolution of contemporary standard-axial field-of-view (SAFOV) PET/CT systems. Such analog PET-systems with photomultiplier tubes (PMT) have lower contrast-to-noise ratios (CNR) and inferior time-of-flight resolution compared to new SiPM-based PET-systems [15, 32]. The introduction of digital whole-body PET/CT systems like LAFOV PET/CT scanners has important advantages compared to previous generation scanners [33]. SiPM-based LAFOV systems have improved sensitivity, higher signal-to-noise ratios and allow for a more precise localization of small target lesions, which is expected to translate into a higher detection rate of small lesions compared to standard PET scanners [19, 26].

In this regard, we here report a patient-based sensitivity for [^68^ Ga]Ga-DOTA-TOC in calcified coronary arteries of 49% with LAFOV PET/CT system. This value is higher compared to that reported by Li et al. [31], wherein also larger arteries were investigated. Differently from previous reports, we observed significant higher [^68^ Ga]Ga-DOTA-TOC uptake in patients with calcifications in more than one vessel. Moreover, increased [^68^ Ga]Ga-DOTA-TOC uptake in coronary arteries correlated with the global- and LAD- CACS. In this context, we report higher TBR compared to the previous report by Rominger et al. (our TBR: 1.65 ± 0.53; Rominger et al.: 1.21 ± 0.30);, which may reflect the noise reduction and increased sensitivity of a LAFOV PET system [9, 10, 26, 34]. However, also differences in the clinical status of their patients may have impacted these results, and this may also explain why TBR in our study was generally lower than reported by Mojtahedi et al. (2.04 ± 1.76). It should be noted that none of our patients had known CAD, while 9.1% of patients in the study by Mojtahedi et al. had prior history of revascularization.

Another important difference to previous reports is that we focused on the detection of increased DOTA-TOC uptake in calcified coronary plaques only. While this choice reflects the retrospective nature of the present study (only a low-dose CT and no CT-based coronary angiography was available), still inflamed plaques with macrocalcifications are important to detect. Previous report showed that the most frequent increased uptake of SST_2_-tracers occurs in calcified plaques. Rominger et al. and Mojtahedi et al. reported that increased uptake was present in 75% of coronary artery calcifications as well as in plaques with high density (> 71 HU) [9, 10]. Likewise, Malmberg et al. showed that high CACS is an independent predictor of increased SUV_max_ with [^64^Cu]Cu-DOTA-TATE [2]. Not less important, plaque calcification is a marker of atherosclerosis, and higher CACS is widely recognized as a robust predictor of MACE [35, 36]. It should be noted that the presence of calcifications in a vascular plaque is a prerequisite for its vulnerability, and there is still a contention regarding the pattern of calcification predictive of higher risk of rupture [37]. Although spotty calcifications have been reported as a potential risk factor for the development of higher degree of inflammation [38], inconsistent findings were seen with regard to largely calcified plaques. Some papers demonstrated that plaque calcification was higher in asymptomatic patients than in symptomatic patients [39], other works showed that a larger calcification volume was associated with a higher prevalence of intra-plaque hemorrhage [40]. Of note, studies specifically investigating the role of a different calcification pattern in coronary plaques with increased uptake of SST_2_-tracers are missing.

Our work expands on the association between calcified plaques with increased uptake of SST_2_-ligands and cardiovascular risk. Stroke occurred more often in patients with [^68^ Ga]Ga-DOTA-TOC uptake in the calcified coronary plaques than in patients without detectable uptake (*p* < 0.01). More importantly, patients with [^68^ Ga]Ga-DOTA-TOC avid calcified plaques had higher rate of all-cause death compared to patients without [^68^ Ga]Ga-DOTA-TOC avid calcified plaques. Our data are in line with prior observations based on the evaluation of both calcified and non-calcified plaques, wherein increased uptake of DOTA-TATE within a coronary plaque was associated with higher rate of MACE independently from other established risk factors [9, 10, 41]. The fact that a similar predictive value also applies to calcified, possibly inflamed plaques in a medium-term follow-up gives confidence to also consider inflamed calcified plaques as determinants of cardiovascular risk. Of note, although all-cause death was used as surrogate for MACE, none of the patients in the study died from oncological reasons, and although a precise cause could not be identified in 9/12 patients, a cardiac origin for the death cannot be ruled out.

In the patients undergoing PRRT, we identified a decrease in [^68^ Ga]Ga-DOTA-TOC uptake within the calcified coronary plaques post-therapy (SUV_max_: 1.46 ± 0.14 pre vs. 0.94 ± 0.19 post, *p*: 0.01). This finding is consistent with previous reports. Schatka et al. also showed that [^68^ Ga]Ga-DOTA-TATE uptake decreases after the PRRT in large vessels [42]. However, we are now able to present first data on the effect of PPRT on vessels as small as coronary arteries. This confirms that the higher sensitivity of LAFOV PET/CT allows for detecting small changes in [^68^ Ga]Ga-DOTA-TOC uptake even in small vessels. Having in mind that [^68^ Ga]Ga-DOTA-TOC uptake is a marker for macrophage activity, we can assume that PRRT may reduce the degree of plaque inflammation [42]. As such, [^68^ Ga]Ga-DOTA-TOC LAFOV PET/CT might be able to identify changes of the plaque inflammation even in small vessels and might be useful to monitor anti-inflammatory therapy.

Since the presence of increased [^68^ Ga]Ga-DOTA-TOC uptake correlates with a worse clinical outcome the degree of uptake may represent the degree of inflammation rather than unspecific activity. As such, we postulate that LAFOV PET/CT might afford the detection of prognostic relevant inflammatory changes in vivo [43]. Reduced tracer uptake after PRRT may also suggest a reduction in the activity of plaque inflammation. While this may serve as a hypothetical therapeutic optional in patients with high cardiovascular risks, it also raises the notion that LAFOV-based [^68^ Ga]Ga-DOTA-TOC PET/CT might serve as a tool for the monitoring of other cardiac therapies. Further studies investigating the influence of PRRT on vascular inflammation and as a tool for therapeutic monitoring are warranted.

Some limitations of our study should be acknowledged. First, as LAFOV systems were recently introduced, our patient sample is small. At the time of investigation, neither ECG-triggered acquisitions nor algorithms to correct for motion artifacts were available for this scanner. Therefore, CT-based coronary angiography (CCTA) was not performed. Thus, as previously mentioned, the retrospective nature of our study prevented us to evaluate the impact of [^68^ Ga]Ga-DOTA-TOC uptake in non-calcified plaques. Additionally, we included oncologic patients referred for a [^68^ Ga]Ga-DOTA-TOC PET/CT and did not select patients with CAD, who might have been treated by a cardiologist in the follow-up period. However, our cohort represents a real world setting where the aim is to characterize cardiac lesions of risk for inflammation as soon as possible. In this regard, it should be noted that patients were not on oncologic therapies other than somatostatin analogs, which exclude a potential bias due to therapeutic regimen. Furthermore, the fact those patients were also not on cardiologic therapy and did not change their therapeutic regimen after PET excludes another potential bias in the evaluation of the prognosis. Most calcified plaques were located in the LAD. The frequency of calcified plaques with [^68^ Ga]Ga-DOTA-TOC uptake apart from LAD was low. Therefore, the prognostic impact of lesion location could not be properly assessed. This is a limitation of our data and should be addressed in further studies. However, the correct identification of significant stenosis (potentially caused by inflamed plaques) in the LAD represents a paramount of importance for therapy decision. In fact, a > 50% stenosis of the proximal LAD with evidence of ischemia is currently considered a robust indication for a successful revascularization [44, 45]. The fact that no correlation between cardiovascular risk factors and the degree of uptake was found differs from what reported in the previous studies [9, 10]. The fact that a different camera system was used may partly explain this discrepancy, as well as the fact that our population did not consist of patients with CAD. But an explanation of this aberrance requires further investigations. Finally, we here considered active only plaques with TBR ≥ 1. Hitherto, no clear cut-off is known for the detection of SST_2_ positive plaques and most evaluation relay on visual interpretation. Previous studies on inflamed plaques considered in the final analysis all lesions irrespective from their TBR [9, 10]. It should be noted that previous works also considered soft plaques. In this regard, the fact that applying our threshold to calcified plaques yielded significant associations with the degree of calcification and with follow-up data gives reliance in considering it adequate on LAFOV PET to identify conceivably inflamed plaques. The degree of such inflammation is then essential to stratify cardiovascular risk.

While additional prospective head-to-head comparisons with, e.g., [^18^F]FDG are needed to support our data and implement LAFOV PET/CT in clinical routine, the results of our study support the concept that LAFOV PET systems may serve as an important tool to identify patients at increased risk of MACE.

## Conclusion

Using [^68^ Ga]Ga-DOTA-TOC as a marker for the M1-macrophage infiltration and subsequent inflammation within calcified coronary plaques in LAFOV PET/CT imaging revealed conceivably inflamed coronary plaques in oncologic patients without history or symptoms of CAD. LAFOV PET could be utilized to assess the inflammation of calcified coronary artery plaques. Patients with higher burden of calcified plaques showed significantly higher [^68^ Ga]Ga-DOTA-TOC uptake, which correlated with higher risk of all-cause death and stroke. If the present results will be confirmed in large prospective trials, [^68^ Ga]Ga-DOTA-TOC LAFOV PET/CT may be considered an useful tool to assess the presence of inflamed, prone-to-rupture coronary plaques, with important advantages in cardiovascular risk stratification.

### Supplementary Information

Below is the link to the electronic supplementary material.

Supplementary file1 (DOCX 201 KB)

## Data Availability

The data are available upon request at the corresponding author’s address.
