# Supplementary material for: Long-axial field-of-view PET/CT for the assessment of inflammation in calcified coronary artery plaques with [68 Ga]Ga-DOTA-TOC
Source: Eur J Nucl Med Mol Imaging. 2023 Sep 23;51(2):422–33. doi: 10.1007/s00259-023-06435-6 (PMC10774639; doi:10.1007/s00259-023-06435-6)
Supplement: Supplementary file 1 — Supplementary file1 (DOCX 201 KB) [file 259_2023_6435_MOESM1_ESM.docx]

**Supplementary Material**

**Table 1:**  Patients` characteristics (sex, height, weight, BMI, age and applied activity) of 108 included patients receiving [^68^Ga]Ga-DOTA-TOC PET/CT are outlined. Additionally primary tumour location, tumour stage and treatment are given.

| Sex | Height  [cm] | Weight  [kg] | BMI  [kg/m^2^] | Age [years] | Activity [MBq] | Primary tumour location | Tumour stage | Treatment |
| --- | --- | --- | --- | --- | --- | --- | --- | --- |
| Male: 67  Female: 41 | 172 (148-184) | 75.5 (39-122) | 25.8±5.6 | 64±15 | 152.2±9.2 | Gastrointestinal: 74  (Stomach/Ileum/Colon)  Pancreas: 20  Paraganglioma: 5  Lung: 3  Testicular: 1  Medullary thyroid cancer: 2  Unknown: 3 | Metastases: 99  No metastases: 9 | No treatment: 11  Somatostatin analogue : 97 (e.g. lanreotide)  PRRT: 4  (Pancreas: 2; Ileum: 2) |


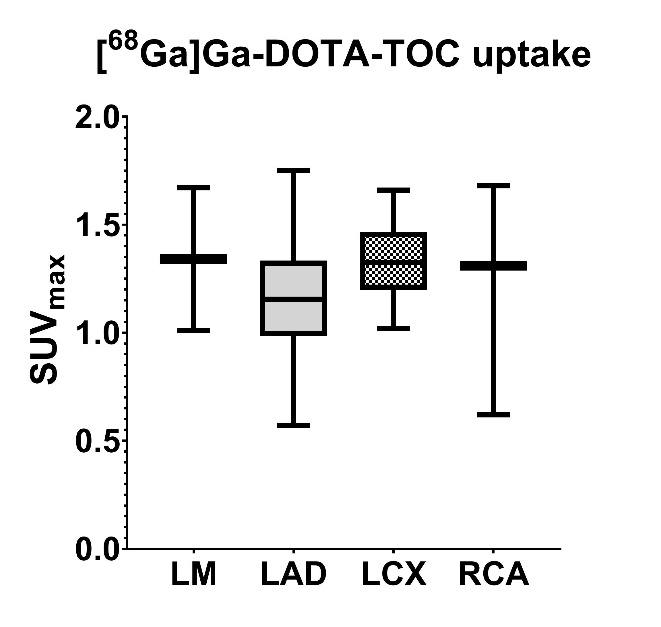


**Figure 1** SUV_max_ in calcified coronary artery plaques per affected vessel.

LM: left main; LAD: left anterior descending, RCX: left circumflex artery; RCA: right coronary artery


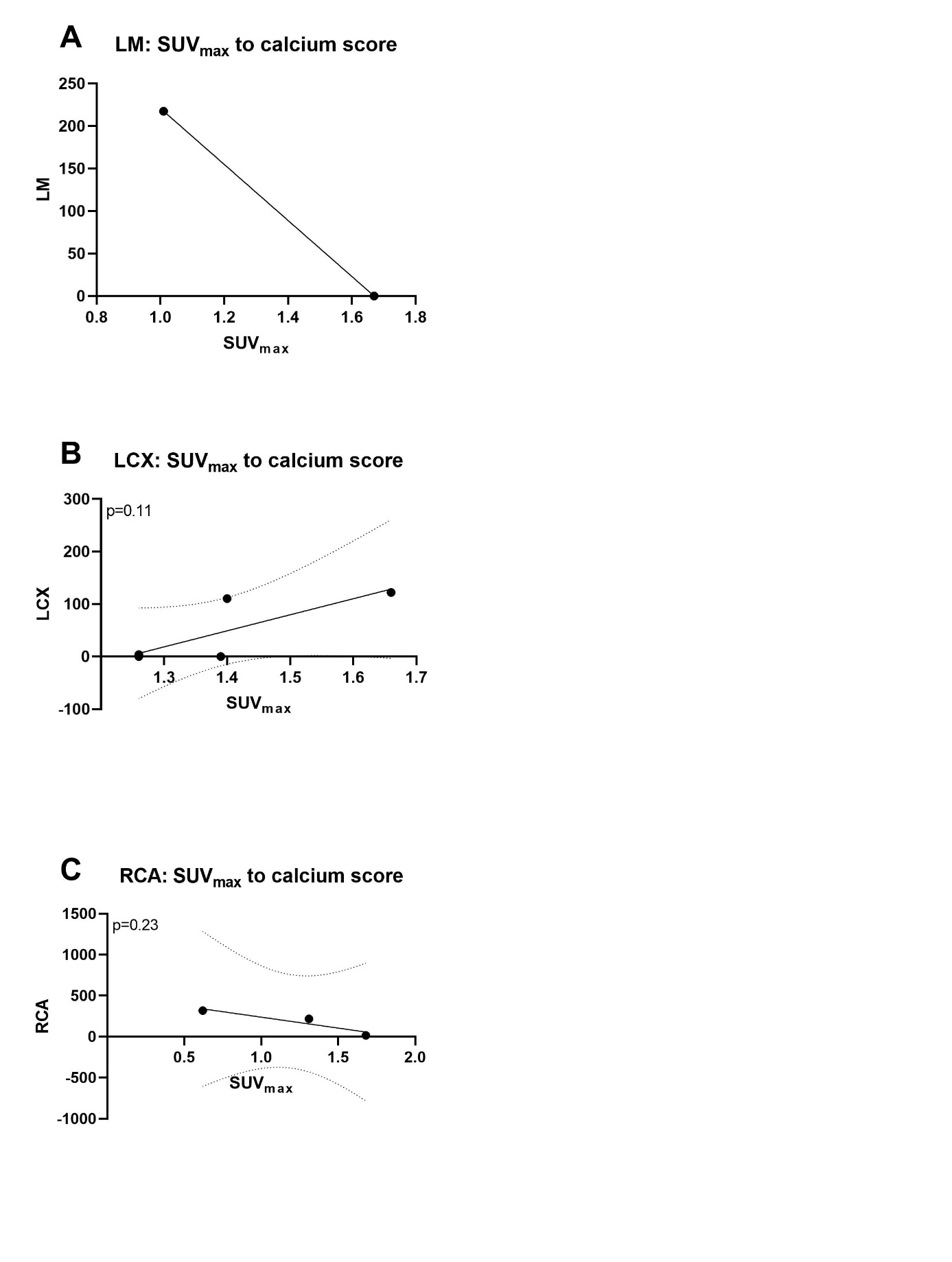


**Figure 2** Linear correlation of Calcium Score and SUV_max_

Indicated are the linear regression models (best fit and standard deviations) of LM (A), LCX (B) and RCA (C) Calcium Score to SUV_max_ ; p-values are given.
